# Twelve-Week Curcumin Supplementation Improves Glucose Homeostasis and Gut Health in Prediabetic Older Adults: A Pilot, Double-Blind, Placebo-Controlled Trial

**DOI:** 10.3390/nu17132164

**Published:** 2025-06-29

**Authors:** Gopal Lamichhane, Tyler J. Godsey, Jing Liu, Rienna Franks, Guolong Zhang, Sam R. Emerson, Yoo Kim

**Affiliations:** 1 Department of Nutritional Sciences, Oklahoma State University, Stillwater, OK 74078, USA; gopal.lamichhane@okstate.edu (G.L.); tylgods@okstate.edu (T.J.G.); rienna.franks@okstate.edu (R.F.); 2 Department of Animal and Food Sciences, Oklahoma State University, Stillwater, OK 74078, USA; jing.liu12@okstate.edu (J.L.); glenn.zhang@okstate.edu (G.Z.)

**Keywords:** curcumin, glucose homeostasis, gut microbiome, HbA1c, type 2 diabetes prevention, geriatric diabetes

## Abstract

**Background**: The prevalence of diabetes increases with age, and food bioactive compounds have shown potential in mitigating its development. This study aimed to evaluate the efficacy of curcumin in preventing type 2 diabetes mellitus (T2DM) in older adults with prediabetes. We hypothesized that curcumin, due to its insulin-sensitizing effects, would help maintain glucose homeostasis, metabolic health, and gut health. **Methods**: This randomized, double-blind, placebo-controlled trial included 28 older adults (aged 60 years or older) with prediabetes or overweight/obesity. Participants were randomly assigned to receive either curcumin (80 mg) or placebo capsules for 12 weeks. The primary outcome measures were glucose homeostasis markers, including hemoglobin A1c (HbA1c), blood glucose, and insulin levels. Secondary outcomes included body composition, body mass index (BMI), body weight, lipid profiles, and gut microbiome composition. Data normality was assessed using the Shapiro–Wilk test, and two-way repeated-measures ANOVA with multiple comparisons was used to find differences between groups and over time. **Results**: A total of 23 participants (age = 66.52 ± 5.76 years; 14 in the curcumin group and 9 in the placebo group) completed the 12-week intervention. HbA1c levels significantly decreased in the curcumin group, whereas levels remained stable in the placebo group. While the curcumin group observed an increase in AST levels, the ALT level was reduced in the placebo group. Both the curcumin and placebo groups showed a reduced ALT/AST ratio by the end of the intervention. Body mass index, lipid profiles, and body composition showed no significant changes over the study period. Gut microbiome analysis revealed no significant changes in alpha diversity or beta diversity of microbiome; however, there was marginal enrichment of beneficial bacteria such as *Bacteroidota* (phylum), *Bacteroidaceae* (family), *Agathobacter*, *Bacteroides*, and *Roseburia* (genera) in the curcumin-supplemented group over time. **Conclusions**: Curcumin supplementation improved or favorably maintained glucose homeostasis and showed modest improvements in beneficial gut microbiota in older adults with prediabetes, potentially reducing the risk of developing diabetes. Long-term studies with larger sample sizes are needed to confirm these findings and determine the clinical relevance of curcumin supplementation for prediabetes prevention.

## 1. Introduction

Diabetes was among the top ten leading causes of death in both the United States and globally in 2023, with approximately 90–95% of cases caused due to type 2 diabetes [1,2]. The burden of diabetes in the elderly population is particularly concerning, as nearly half of individuals with type 2 diabetes are aged 65 or older [3]. According to the Centers for Disease Control and Prevention (CDC), around 15% of U.S. adults aged 18 and older have diabetes, a rate that rises to nearly 30% in those aged 65 and above [4]. The management of type 2 diabetes in older adults is further complicated by population heterogeneity, the presence of comorbidities, increased susceptibility to hyperglycemia, frailty, and greater dependence on caregiving [3]. As the global population ages due to advancements in healthcare, preventing type 2 diabetes in the elderly has become an urgent public health priority [5].

Currently, there is no cure for type 2 diabetes, and the most common approach involves lifelong use of hypoglycemic medications and/or insulin therapy to manage the disease [6]. Therefore, identifying risk factors early and implementing preventive strategies is critical to reducing the disease burden. Lifestyle interventions, such as those developed by the CDC-led National Diabetes Prevention Program, have proven effectiveness in preventing or delaying the onset of diabetes among at-risk individuals [7,8]. Early management of body weight and prediabetes is key to reducing the incidence of diabetes and its complications, thereby improving quality of life [7]. This is because obesity is a major contributor to type 2 diabetes, as both conditions share common metabolic defects and often escalate in parallel. Excess adiposity promotes insulin resistance and impairs insulin secretion, ultimately leading to the development of diabetes [9]. Therefore, anti-obesity medications like GLP1 agonists and orlistat are widely used in the management of diabetes and its complications [10,11]. Another promising preventive strategy is the use of dietary interventions (caloric restriction and food bioactive compounds) targeting individuals with prediabetes before progression to full-blown diabetes [12,13,14]. However, the success of this approach depends on timely risk identification and the efficacy of the intervention. Importantly, there is a significant research gap in diabetes prevention studies specifically focused on older adults, a population with both the highest prevalence of diabetes and the fastest-growing demographic segment [15].

Emerging evidence indicates that gut microbiota plays a crucial role in the development and progression of diabetes. Alterations in microbial composition have been linked to glycemic control and insulin resistance, highlighting the relevance of gut health in diabetes management [16]. Individuals with prediabetes or type 2 diabetes often show a reduced abundance and functional potential of butyrate-producing bacteria [16], along with decreased microbial diversity compared to non-diabetic individuals [17,18,19]. These diabetes-associated changes in the gut microbiome may compromise gut barrier integrity and increase the translocation of harmful substances such as lipopolysaccharides (LPS) into systemic circulation, thereby exacerbating inflammation and impairing glucose homeostasis [20,21,22]. Hence, modulating gut microbial populations through dietary strategies provides a compelling avenue for diabetes prevention and management.

Curcumin, a bioactive polyphenolic compound derived from turmeric (*Curcuma longa*), has garnered attention for its potential health benefits, particularly in the context of metabolic disorders. Several studies suggest that curcumin can positively influence glucose metabolism and gut microbiota composition, both critical factors in the context of diabetes management [23,24,25,26]. However, most intervention studies to date have focused on younger or mixed-age populations, leaving a gap in our understanding of curcumin’s efficacy specifically in older adults [24,26,27,28]. Our previous preclinical studies in an aged mouse model suggested improved insulin signaling and metabolic health benefits under diet-induced obesity [29,30,31]. To address this gap and validate our preclinical findings in humans, the current study aims to evaluate the effects of a 12-week curcumin supplementation regimen on glucose regulation and gut health in elderly individuals at high risk for type 2 diabetes. We hypothesized that curcumin, through its metabolic and microbiome-modulating properties, would ameliorate hyperglycemia and improve metabolic and gut health in this vulnerable population.

## 2. Materials and Methods

### 2.1. Participants and Study Design

This 12-week, double-blind, randomized, placebo-controlled trial was conducted at the research facilities of the Department of Nutritional Sciences at Oklahoma State University in Stillwater, Oklahoma. The study protocol was approved by the Institutional Review Board (IRB) of Oklahoma State University (IRB application number: IRB-23-7; approved on 16 March 2023) and registered at ClinicalTrials.gov (NCT06984640; Registration date: 14 May 2025). The research was conducted in compliance with the ethical principles outlined in the Declaration of Helsinki. Additionally, the study adhered to Good Clinical Practice (GCP) guidelines and followed the CONSORT checklist for reporting randomized trials (Supplementary File S1).

Potential participants aged 60 years and older were recruited via “mass email” and flyers distributed at Oklahoma State University in Stillwater between August 2023 and April 2024. All participants were given the opportunity to review and discuss an informed consent form detailing the potential benefits and risks associated with participation. To be eligible, participants were required to meet at least one of the following criteria: (a) prediabetes, defined as a fasting blood glucose level of 100–125 mg/dL or an HbA1c of 5.7–6.4%, or (b) a body mass index (BMI) ≥ 25 Kg/m^2^. Exclusion criteria included: (a) a preexisting cardiometabolic condition such as diabetes, (b) liver disease, (c) dementia, (d) tobacco use within one year prior to the study, and (e) use of medications targeting diabetes or blood lipids. Eligible participants were randomly assigned to receive either a placebo or a curcumin capsule daily for 12 weeks. Each capsule contained 400 mg of a patented lipophilic matrix, delivering 80 mg of curcumin or dextrin with 0.05% tartrazine (a yellow food coloring). Longvida^®^ is a trademark of Vendure Sciences, Noblesville, IN, USA. The low dose of curcumin was chosen as a previous study in older adults showed gastrointestinal side effects under high doses [32], while the selected formulation was well tolerated by the participant without side effects [33]. The formula was a patented optimized dose with higher solubility to deliver a high amount to systemic circulation, overcoming the bioavailability issue of curcumin [34]. Treatment capsules were dispensed in bottles after baseline measurements were completed, with instructions to take one capsule per day after a meal. Participant compliance was monitored at weeks 6 and 12 by reviewing logbooks documenting supplement intake and by counting the remaining capsules at the end of the study. Participants were instructed to report any adverse effects or discomfort via email, text message, or phone call.

### 2.2. Sample Size, Randomization, and Allocation

To accommodate a potential dropout rate of 10% to 20%, we targeted enrollment of 15 to 17 participants per group. A post hoc power analysis was conducted for a 2-sample paired *t*-test using the GraphPad Prism statistical program (version 10.2.2). The analysis determined that a group size of 15 participants would provide over 50% power to detect a mean of −0.2500 difference in HbA1c between the curcumin group, applying a standard deviation difference of 0.4433 and a significance level (alpha) of 0.05 (two-tailed). Participant allocation was randomized using a computer-generated sequence. All supplements and placebos were coded with anonymous identification numbers by a research advisor who was not involved in participant recruitment or outcome assessments. Other researchers distributed the supplement bottles to participants without knowledge of group assignments, ensuring that both the investigators and participants remained blinded to the treatment allocation throughout the study. The randomized code was only revealed by the advisor after the study was completed, at which point the data were unblinded for analysis. Neither the participants nor the investigator had access to the randomization assignments during the intervention period, thereby maintaining the integrity of the double-blind study design.

### 2.3. Data Collection and Outcomes

The primary outcome measures of this study were HbA1c levels and fasting blood glucose levels. Secondary outcomes included body weight, waist circumference, body composition, lipid profile (triglycerides, total cholesterol, low-density lipoprotein (LDL), high-density lipoprotein (HDL), and very low-density lipoprotein (VLDL)), hepatic enzymes (aspartate aminotransferase (AST) and alanine aminotransferase (ALT)), lactate levels, blood pressure, and changes in the gut microbiome composition. Participants were assessed for overnight fasting blood glucose, HbA1c, body weight, and height to determine eligibility. If they met the inclusion criteria, the following data were subsequently collected: (a) lipid profile (triglycerides, total cholesterol, LDL, HDL, and VLDL), (b) hepatic enzymes (AST and ALT), (c) Lactate levels, (d) 24-h dietary recall, (e) medical history questionnaire, (f) body composition using dual-energy X-ray absorptiometry (DXA (Hologic Horizon A; Marlborough, MA, USA)), and (g) blood pressure. Participants were also asked to provide a fresh stool sample using a stool collection kit, which was stored on ice packs immediately after collection and later frozen at −80 °C for microbiome analysis. Following metabolic assessment with whole blood, the remaining serum samples were also stored at −80 °C for further analysis, including serum insulin levels. Participants were randomly assigned to their respective groups after the collection of baseline stool samples. All measurements were repeated at the end of the 12-week intervention.

### 2.4. Analysis of HbA1c, Lactate, Lipid Panel, Hepatic Enzymes, and Serum Insulin Levels

The HbA1c level was measured in DCA Vantage^TM^ Analyzer from SIEMENS (Malvern, PA, USA) using DCA HbA1c Reagent Kit (ref no. 11645002). Blood lactate level was measured using a Lactate Plus lactate test strip by Nova Biomedical (Waltham, MA, USA; ref no. 40813) using a Lactate Plus Meter from Nova Biomedical (ref no. 62624). BMI was calculated using body weight (measured using SECA medical body composition analyzer) and height. Waist circumference was measured by placing tape horizontally at the level of the navel. Blood pressure was measured (3 times) by using a blood pressure monitor (Model BP7350) from Omron Healthcare Inc. (Lake Forest, IL, USA). Metabolic outcomes, including glucose, lipids, and hepatic enzymes, were measured with a Piccolo Xpress Clinical Chemistry analyzer (Abbott, Chicago, IL, USA) using Lipid Panel Plus reagent discs. Glucose, ALT, AST, total cholesterol, HDL, and triglycerides were measured, while LDL and VLDL were calculated. Serum insulin levels were measured using an Insulin ELISA kit (Ref: 4212508; Thermo Fisher Scientific, San Diego, CA, USA) according to the manufacturer’s protocol.

### 2.5. Gut Microbiome Analysis

Genomic DNA was isolated from stool samples using the QIAamp^®^ Fast DNA Stool Mini Kit (QIAGEN, Hilden, Germany) and submitted to Novogene for 16S rRNA gene sequencing targeting the V3-V4 region. Sequencing data were deposited in the National Center for Biotechnology Information (NCBI) Sequence Read Archive (SRA) and are accessible under the BioProject accession number PRJNA1217483. The paired-end 16S rRNA V3-V4 sequencing reads were processed and analyzed using QIIME 2 (v. 2020.11). Prior to downstream analysis, adaptor, barcode, and primer sequences were removed using the “Cutadapt” plugin in QIIME 2. Forward and reverse reads were then merged, and quality filtering was applied to filter out low-quality reads. The Deblur algorithm (v. 2022.8.0) was used to denoise the data and generate Amplicon Sequence Variants (ASVs). The taxonomic classification of ASVs was performed using the Ribosomal Database Project (RDP) 16S rRNA training set (v. 18) with the Bayesian classifier [35]. ASVs with a bootstrap confidence score less than 80% were assigned to the last confidently identified taxonomic level, followed by “_unclassified.” The top 30 ASVs, along with all differentially enriched ASVs, were further validated and reclassified, if necessary, using the most recent EzBioCloud 16S database (v. 2023.08.023). ASVs present in fewer than 5% of samples were excluded from further analysis. Differential enrichment of bacterial ASVs between different experimental groups was assessed using Linear Discriminant Analysis (LDA) Effect Size (LEfSe). Statistical significance was determined using an all-against-all multiclass analysis, with a significance of *p*-value threshold of < 0.05 and a logarithmic LDA score threshold of 2.5.

### 2.6. Statistical Analysis

Statistical analyses were conducted using GraphPad Prism (version 10.2.2). The normality of data distribution was assessed using the Shapiro–Wilk test. For normally distributed data, two-way repeated-measures ANOVA was applied to evaluate differences between groups and over time, followed by Bonferroni correction for post hoc multiple comparisons in ANOVA. Log transformation was carried out for data not following normal distribution before performing the ANOVA function. Categorical data were analyzed using the chi-square test. For gut microbiota analysis, differences in microbiome diversity between groups, before and after the intervention, were identified using LEfSe.

## 3. Results

### 3.1. Participants’ Characteristics and Baseline Measurement

**Figure 1:**
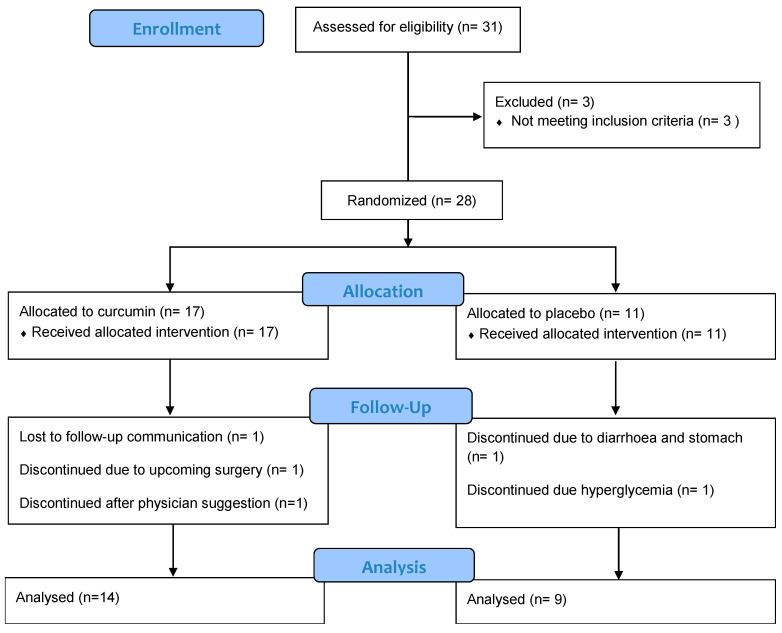
CONSORT diagram illustrating the progression of participants through each stage of the study, including screening, randomization, allocation to intervention groups, follow-up, and analysis.

Among the 31 individuals screened for eligibility, 28 participants met the inclusion criteria and were randomized to either the curcumin group (*n* = 17) or the placebo group (*n* = 11). A total of 23 participants completed the 12-week intervention: 14 in the curcumin group and 9 in the placebo group (Figure 1). All data analyses were performed on these 23 participants who completed the study. An unpaired *t*-test was used to compare baseline characteristics between the curcumin and placebo groups. The results indicated appropriate randomization, with no significant differences between groups on most parameters (*p* > 0.05), except for systolic blood pressure and cholesterol (Table 1). The baseline difference in these variables should be considered while interpreting the measures. Table 1 summarizes the baseline characteristics of the participants who completed the study.

There were five dropouts in total: three from the curcumin group and two from the placebo group. In the curcumin group, one participant withdrew after beginning antihypertensive medication, following her physician’s recommendation (baseline blood pressure: 150/97 mmHg). Another participant dropped out due to a scheduled surgery. The third participant discontinued communication with research staff after the 6-week mid-intervention appointment. In the placebo group, one participant withdrew due to diarrhea and stomach discomfort. Another discontinued participation due to elevated blood glucose levels. An additional participant, who completed the study, reported constipation that resolved after a few weeks post-study. Overall, no serious adverse events related to curcumin supplementation were reported during the trial. The study was terminated in April 2024, as per the project timeline.

### 3.2. Effect of Curcumin on Glucose Homeostasis, Lipid Profile, and Hepatic Enzymes

Although no significant group × time interaction was observed (*p* = 0.217), the curcumin-supplemented group showed a significant reduction in HbA1c levels (*p* = 0.044, 95% CI: 0.006 to 0.494), while levels remained stable in the placebo group (*p* > 0.999, 95% CI: −0.260 to 0.348). In contrast, the fasting blood glucose levels remained the same in both the curcumin (*p* = 0.154, 95% CI: −7.547 to 0.976) and placebo group (*p* > 0.999, 95% CI: −5.537 to 5.093). Although there was no notable change in the cholesterol and LDL levels of the curcumin group, the placebo group exhibited marginally higher Q1 and Q3 for cholesterol level (*p* = 0.101). No significant changes were observed in serum triglyceride, VLDL, HDL, and lactate levels in either group over time. For hepatic enzymes, mean ALT levels significantly decreased from 30 (28–38) to 26 (26–30) (*p* = 0.028) in the placebo group, whereas ALT was not reduced significantly in the curcumin group (pre-intervention: 32.5 (22.75–38.5) to post intervention: 27 (22.75–33.75) (*p* = 0.224)). Conversely, AST levels significantly increased in the curcumin group from 30.5 (24.25–38) to 36 (25.5–48.5) (*p* = 0.028), while mean AST level was not increased significantly in the placebo group (pre-intervention: 29 (25–33) to post intervention: 31 (30–35) (*p* = 0.191)). However, the values of the ALT/AST ratio significantly decreased in both groups (curcumin: *p* = 0.002, 95% CI: 0.087 to 0.362; placebo: *p* < 0.001, 95% CI: 0.145 to 0.492) following the intervention period. There were no significant changes in serum insulin or HOMA-IR in either group after the 12-week intervention (Table 2).

### 3.3. Effect of Curcumin on Nutrient Intake, Arthropometric Parameters, and Body Composition

Nutrient intake was assessed using a 24-h dietary recall. No significant change in dietary pattern was observed in participants in both groups and over time (Table 3). Although there was some pattern, such as reduced total calorie, protein, fat, and cholesterol intake in the curcumin group and lower total calorie, fat, saturated fat, trans fat, and increased cholesterol intake in the placebo group, none reached a significant level due to higher variance between subjects.

Table 4 shows the anthropometric and body composition data before and after the 12-week intervention for both the curcumin and placebo groups. At the end of the intervention period, systolic blood pressure remained significantly higher in the curcumin-supplemented group compared to the placebo group (*p* = 0.007, 95% CI: −34.15 to −4.746). However, there were no statistically significant differences between or within groups for any other parameters, including body weight, BMI, waist circumference, diastolic blood pressure, bone mineral content (BMC), bone mineral density (BMD), fat mass, lean mass, and percentage fat mass.

### 3.4. Effect of Curcumin on Gut Microbiota Diversity and Composition

**Figure 2:**
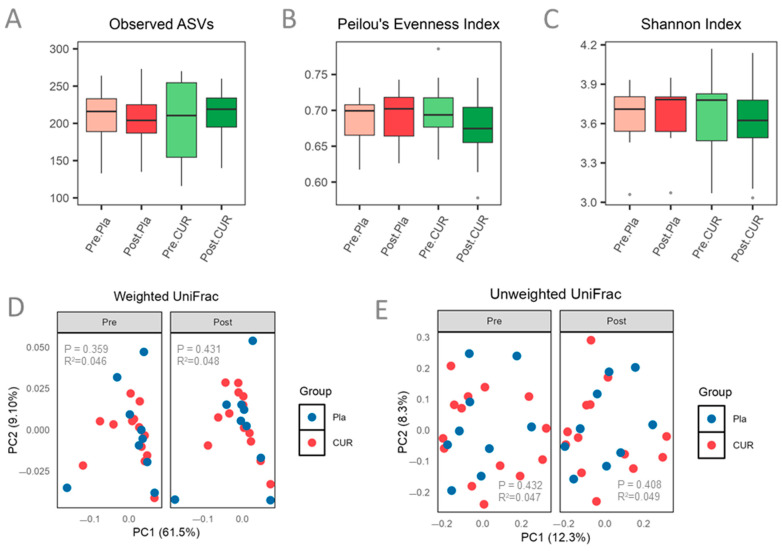
No significant changes in alpha or beta diversity of the gut microbiome were observed following the 12-week intervention: (**A**) observed ASVs, (**B**) Pielou’s Evenness Index, (**C**) Shannon Index, (**D**) Weighted UniFrac distance, and (**E**) unweighted UniFrac distance for the curcumin and placebo groups. Pre: before supplementation; Post: after intervention; Pla: placebo; and CUR: curcumin.

A total of 4,670,073 sequencing reads were initially obtained. After quality control procedures, 4,444,073 high-quality reads remained. These reads were denoised using Deblur and clustered into 2712 ASVs. After filtering out ASVs present in fewer than 5% of the samples, 839 ASVs were retained for downstream analysis. Alpha diversity was assessed using observed ASVs, Pielou’s Evenness Index, and Shannon Index, whereas beta diversity was evaluated using both weighted and unweighted UniFrac distance metrics. Changes in alpha diversity (observed ASVs, Pielou’s Evenness Index, and Shannon index) are presented in Figure 2A–C, and beta diversity results (weighted and unweighted UniFrac distances) are presented in Figure 2D, E. No significant differences in alpha or beta diversity were observed between or within groups as a result of the 12-week intervention.

**Figure 3:**
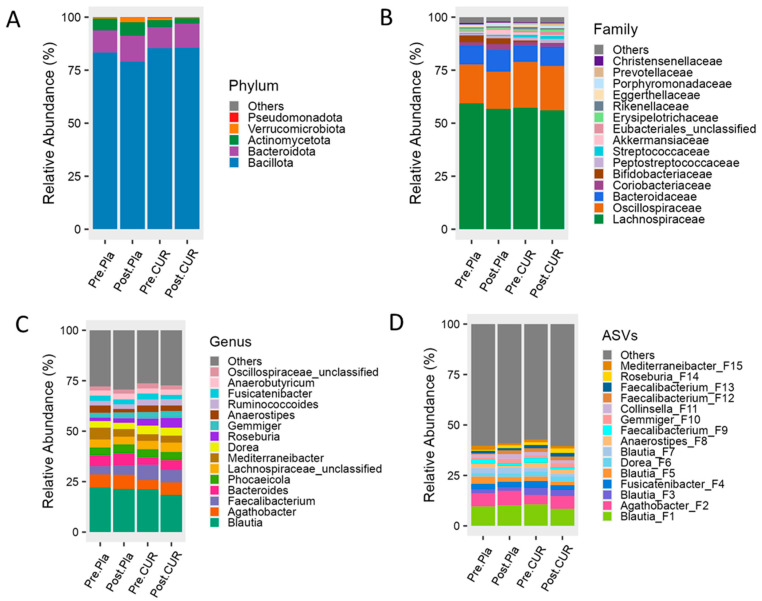
Twelve weeks of curcumin supplementation modestly alters gut microbial composition in older adults. The figure represents microbial composition at the (**A**) phylum, (**B**) family, (**C**) genus, and (**D**) ASVs levels. Pre: before supplementation; Post: after intervention; Pla: placebo, and CUR: curcumin.

At the phylum level, *Bacillota*, *Bacteroidota*, and *Actinobacteriota* were dominant in both the curcumin and placebo groups before and after the intervention. At the family level, *Lachnospiraceae*, *Oscillospiraceae*, *Bacteroidaceae*, and *Coriobacteriaceae* were most prevalent. The predominant genera across all groups included *Blautia*, *Agathobacter*, *Faecalibacterium*, *Bacteroides*, *Phocaeicioia*, *Lachnospiraceae_unclassified*, *Mediterraneibacter*, *Dorea*, and *Roseburia*. While the curcumin group showed a modest increase in the relative abundance of potentially beneficial bacteria, including *Bacteroidota* (phylum), *Bacteroidaceae* (family), and *Agathobacter*, *Bacteroides*, and *Roseburia* (genera), after 12 weeks (Figure 3A–D), these differences were not statistically significant under LEfSe.

LEfSe was used to identify differentially abundant ASVs within groups over time. However, no statistically significant differences in bacterial ASV profiles were observed within groups over the 12-week intervention period.

## 4. Discussion

The measurement of HbA1c is widely recognized as one of the most reliable and robust indicators of long-term glycemic control [36]. This is because HbA1c reflects the average of hundreds—potentially thousands—of fasting glucose levels and also captures postprandial glucose spikes [37]. In the current study, we observed a significant reduction in HbA1c levels among elderly participants with prediabetes who received low-dose curcumin supplementation (80 mg/day) over a 12-week period. Although the group × time interaction was not statistically significant, the within-group improvement in the curcumin group suggests a potential benefit that warrants further investigation. This finding is noteworthy as it supports previous clinical trials that demonstrated curcumin’s potential to lower HbA1c levels in individuals with newly diagnosed diabetes (age 35+), although those studies used much higher doses (e.g., 1500 mg/day) [24]. Our results suggest that even a low dose of curcumin may effectively improve glycemic control in elderly populations, which could enhance patient compliance and minimize the risk of side effects commonly associated with higher doses. However, despite the improvement in HbA1c in the curcumin group, we were not able to observe a significant difference in fasting blood glucose levels in the curcumin group over the intervention period. This finding is in contrast to prior studies reporting improved fasting blood glucose with curcumin supplementation [27,28]. The discrepancy may be attributed to two key factors: the relatively low curcumin dose used in our study and the lower reliability of single-timepoint fasting glucose measurements, which can be influenced by various physiological and environmental factors [37]. No significant changes in body lipid profile, body weight, BMI, waist circumference, or body composition were observed in either group. This suggests that curcumin’s effect on HbA1c may be mediated through insulin-sensitizing properties independent of weight loss or changes in body composition [38]. It is possible that higher doses or longer durations of curcumin supplementation may be required to elicit measurable changes in these parameters [24,39].

Although AST and ALT are considered limited in reliability as biomarkers of hepatic health, they remain widely used due to their noninvasive and cost-effective nature [40]. In this study, we observed a significant increase in AST levels in the curcumin group. Conversely, ALT levels decreased in the placebo group. These results differ from previous meta-analyses that have reported significant reductions in both AST and ALT with curcumin supplementation [41]. The discrepancy may be explained by differences in age groups, curcumin dosage, and formulation. Notably, the ALT/AST ratio, an emerging marker correlated with hepatic steatosis and liver fibrosis, was decreased in both groups [42]. Further studies incorporating larger sample sizes and more comprehensive hepatic biomarkers are needed to clarify curcumin’s effects on liver health in older adults.

Emerging evidence highlights the significant role of the gut microbiota in regulating key metabolic functions, particularly lipid and glucose homeostasis [43,44,45]. Disruptions in microbial composition and function are increasingly associated with the development of metabolic disorders such as dyslipidemia and type 2 diabetes [43,45,46]. This connection is largely mediated through microbial metabolites, including short-chain fatty acids (SCFAs), secondary bile acids, and lipopolysaccharides, which modulate insulin sensitivity, lipid metabolism, and systemic inflammatory responses [46,47,48]. Regarding gut microbiota, prior studies have shown curcumin to modulate microbiome composition and increase the abundance of beneficial bacteria linked to metabolic health [26,49]. In our study, we observed a modest, non-significant increase in beneficial taxa—specifically *Bacteroidota* (phylum), *Bacteroidaceae* (family), and *Agathobacter*, *Bacteroides*, and *Roseburia* (genera)—in the curcumin group after 12 weeks. However, no statistically significant changes in alpha or beta diversity or in taxonomic composition were observed between or within the groups. This lack of significance may be due to the low number of participants in the study and higher inter-individual variability in the gut microbiota of older adults than in younger populations [50,51]. Further studies using higher doses of curcumin in larger numbers of participants for extended intervention periods and controlled dietary regimens may be more effective in demonstrating gut microbiota alterations [25,26,52].

Several limitations should be considered when interpreting these findings. First, we did not reach our targeted sample size necessary to give sufficient power due to project timeline constraints, which may have limited the study’s statistical power. Participants’ recruitment relied on mass emailing, potentially excluding individuals without internet access and limiting generalizability to broader populations. Additionally, the unequal distribution of gender and sample size between the curcumin and placebo groups introduces the possibility of bias. Baseline differences in potential confounders, such as systolic blood pressure and cholesterol levels, may have influenced the outcome measures. Heterogeneity in participant characteristics, including body weight, BMI, and baseline glucose status, may also have impacted the study results. Importantly, the absence of a statistically significant group × time interaction limits our ability to attribute the observed HbA1c reduction solely to curcumin supplementation. These factors should be taken into account when interpreting the results. Future studies with larger, more balanced, and diverse populations over a longer period of time are warranted.

## 5. Conclusions

In conclusion, we observed improved glucose homeostasis and modest enrichment of beneficial gut microbiota in older adults under curcumin supplementation. Although the results are promising, further research involving larger sample sizes, longer intervention periods, and diverse populations is needed to establish definitive clinical guidelines for curcumin supplementation.

## Figures and Tables

**Table 1:** Baseline characteristics of participants in the curcumin and placebo groups.

Measures			All Participants (*n* = 23)	Curcumin (*n* = 14)	Placebo (*n* = 9)	*p*-Value
Demographic data						
Age (Years)			66.52 ± 5.76	65.5 (61.25–72)	67 (62–69)	0.914 ^b^
Gender	Male		6	4	2	0.735 ^c^
	Female		17	10	7	
Weight (Kg)			85.44 ± 19.81	88.56 ± 21.94	80.59 ± 14.69	0.369 ^a^
BMI (Kg/m^2^)			31.25 ± 6.45	32.36 ± 7.33	29.52 ± 4.25	0.325 ^a^
Waist circumference (cm)			100.72 ± 13.34	103.14 ± 15.23	96.94 ± 8.38	0.298 ^a^
Blood pressure (mmHg)		Systolic	126.87 ± 15.61	133.88 ± 13.74	115.96 ± 11.55	0.005 ^a^
		Diastolic	81.75 ± 9.80	81 (75–84.75)	81 (73.67–81.67)	0.496 ^b^
Blood glucose (mg/dL)			106.43 ± 7.93	107.07 ± 9.21	105.44 ± 5.21	0.649 ^a^
HbA1c (%)			5.61 ± 0.40	5.66 ± 0.47	5.52 ± 0.22	0.429 ^a^
Lactate (mmol/L)			1.28 ± 0.47	1.38 ± 0.47	1.10 ± 0.42	0.203 ^a^
Triglyceride (mg/dL)			123.26 ± 69.80	84.5 (72.25–157.75)	98 (78–151)	0.793 ^b^
Cholesterol (mg/dL)			185.09 ± 40.57	198.86 ± 38.24	163.67 ± 34.32	0.044 ^a^
HDL (mg/dL)			61.83 ± 12.70	65.5 (56.25–68.5)	60 (58–63)	0.231 ^b^
LDL (mg/dL)			98.43 ± 32.91	109.21 ± 29.62	81.67 ± 30.68	0.053 ^a^
VLDL (mg/dL)			24.23 ± 14.23	17 (14.25–31.75)	19.5 (15.75–23.25)	0.934 ^b^
ALT (U/L)			32.82 ± 9.43	32.79 ± 10.82	32.89 ± 6.71	0.981 ^a^
AST (U/L)			30.65 ± 6.96	31.00 ± 7.82	30.11 ± 5.32	0.778 ^a^
Serum Insulin level (µIU/mL)			9.15 ± 6.74	5.93 (4.58–8.32)	10.50 (7.45–10.68)	0.125 ^b^
HOMA-IR			2.40 ± 1.74	1.67 (1.12–2.18)	2.79 (2.03–2.90)	0.125 ^b^
Body composition						
BMC (Kg)			2.22 ± 0.46	2.28 ± 0.45	2.14 ± 0.46	0.481 ^a^
BMD (g/cm^2^)			1.08 ± 0.13	1.10 ± 0.12	1.06 ± 0.14	0.480 ^a^
Fat mass (Kg)			33.24 ± 11.12	34.71 ± 12.96	30.95 ± 6.78	0.452 ^a^
Lean mass (Kg)			50.78 ± 12.30	48 (44.11–54.97)	50.09 (41.02–54.18)	0.600 ^b^
% fat			38.22 ± 7.33	38.38 ± 8.44	37.98 ± 5.13	0.904 ^a^

All the data are reported as either mean ± standard deviation (for normally distributed data) or median (Q1-Q3) (for data not distributed normally). BMI: body mass index, HbA1c: hemoglobin A1c; HDL: high-density lipoprotein; LDL: low-density lipoprotein; VLDL: very low-density lipoprotein; ALT: alanine aminotransferase; AST: aspartame aminotransferase; BMC: bone mineral content; BMD: bone mineral density, ^a^ unpaired *t*-test, ^b^ Mann–Whitney test, ^c^ chi square test.

**Table 2:** Effects of 12-week curcumin supplementation on glucose homeostasis, lipid profile, and hepatic enzyme levels in elderly participants.

Measures	Curcumin (*n* = 14)			Placebo (*n* = 9)			*p*-Value Post-Intervention Between Groups	*p*-Value Interaction
	Pre-Intervention	Post-Intervention	*p*-Value Within Group	Pre-Intervention	Post-Intervention	*p*-Value Within Group		
Blood glucose (mg/dL)	107.07 ± 9.21	110.36 ± 12.73	0.154	105.44 ± 5.21	105.67 ± 7.56	>0.999	0.559	0.290
HbA1c (%)	5.66 ± 0.47	5.41 ± 0.45	0.044	5.52 ± 0.22	5.48 ± 0.20	>0.999	>0.999	0.217
Lactate (mmol/L)	1.30 (1.03–1.75)	1.20 (0.90–1.40)	0.893	0.90 (0.85–1.40)	1.15 (0.93–1.80)	0.685	>0.999	0.228
Triglyceride (mg/dL)	84.50 (72.25–157.75)	92.50 (73.75–157.50)	>0.999	98 (78–151)	109 (99–138)	0.609	>0.999	0.440
Cholesterol (mg/dL)	187.5 (171–218.5)	190 (172.75–197.5)	>0.999	176 (145–190)	177 (164–200)	0.101	0.489	0.112
HDL (mg/dL)	65.5 (56.25–68.50)	66.50 (59.5–68.75)	>0.999	60 (58–63)	62 (61–65)	0.306	>0.999	0.233
LDL (mg/dL)	109.21 ± 29.62	108.86 ± 2 5.66	0.990	81.67 ± 30.68	93.22 ± 28.94	>0.999	>0.999	0.965
VLDL (mg/dL)	17 (14.25–31.75)	18.5 (15–31.25)	>0.999	19.5 (15.75–23.25)	17 (14.25–31.75)	0.332	>0.999	0.291
ALT (U/L)	32.5 (22.75–38.5)	27 (22.75–33.75)	0.224	30 (28–38)	26 (26–30)	0.028	>0.999	0.404
AST (U/L)	30.5 (24.25–38)	36 (25.5–48.5)	0.028	29 (25–33)	31 (30–35)	0.191	>0.999	0.758
ALT/AST ratio	1.05 ± 0.19	0.80 ± 0.21	0.002	1.11 ± 0.24	0.84 ± 0.28	<0.001	>0.999	0.302
Serum Insulin level (µIU/mL)	5.93 (4.58–8.32)	6.93 (3.63–12.62)	>0.999	10.50 (7.45–10.68)	8.45 (5.73–12.85)	>0.999	>0.999	0.704
HOMA-IR	1.67 (1.12–2.18)	1.85 (0.95–3.57)	>0.999	2.79 (0.02–2.90)	2.15 (1.50–3.57)	>0.999	>0.999	0.648

All the data are reported as either mean ± standard deviation (for normally distributed data) or median (Q1–Q3) (for data not distributed normally). HbA1c: hemoglobin A1c; HDL: high-density lipoprotein; LDL: low-density lipoprotein; VLDL: very low-density lipoprotein; ALT: alanine aminotransferase; AST: aspartame aminotransferase; HOMA-IR: Homeostatic Model Assessment of Insulin Resistance.

**Table 3:** Dietary patterns of participants before and at the end of the intervention period.

Measures	Curcumin (*n* = 14)			Placebo (*n* = 9)			*p*-Value Post-Intervention Between Groups	*p*-Value Interaction
	Pre-Intervention	Post-Intervention	*p*-Value Within Group	Pre-Intervention	Post-Intervention	*p*-Value Within Group		
Total calories (kcal)	2075 (1741.25–2348.22)	1463.47 (1196.32–2413.83)	0.545	1977 (1512.25–2160.44)	1549.8 (1354.39–2009.28)	0.486	>0.999	0.818
Calories from fat (kcal)	785.27 (491.61–815.67)	595.98 (339.36–809.84)	>0.999	728.06 (544.02–1011.54)	532.2 (458.59–597.07)	0.207	0.510	0.325
Protein (g)	90.17 (72.50–143.13)	67.14 (43.67–100.99)	0.169	72.87 (65.85–85.47)	81.53 (69.75–96.63)	0.890	0.999	0.444
Carbohydrates (g)	212.27 ± 68.21	203.29 ± 74.73	>0.999	215.70 ± 55.79	183.05 ± 57.91	0.714	0.996	0.600
Total dietary fiber (g)	13.91 (8.79–22.85)	13.89 (8.57–20.75)	0.989	19.5 (16.52–25.41)	14.57 (5.64–22.6)	0.903	0.721	0.820
Sugar (g)	87.24 ± 33.52	87.35 ± 51.50	>0.999	94.73 ± 37.41	75.20 ± 42.34	0.649	>0.999	0.438
Fat (g)	87.45 (54.62–90.78)	66.22 (37.71–89.98)	0.850	80.9 (60.45–112.39)	59.13 (50.95–66.34)	0.196	0.509	0.325
Saturated fat (g)	28.22 ± 10.16	28.15 ± 19.04	>0.999	25.35 ± 13.08	17.95 ± 7.59	0.579	0.205	0.410
Trans-fatty acid (g)	0.29 (0.09–0.61)	0.34 (0.09–1.57)	0.708	0.26 (0.07–0.52)	0.07 (0–0.25)	0.856	0.971	0.960
Cholesterol (mg)	335.99 (183.87–514.02)	244.39 (192.45–482.09)	0.917	122.16 (76.15–214.83)	260.75 (115.05–397.77)	0.251	>0.999	0.159

All the data are reported as either mean ± standard deviation (for normally distributed data) or median (Q1–Q3) (for data not distributed normally).

**Table 4:** Effects of 12-week curcumin supplementation on anthropometric parameters in elderly participants.

Measures		Curcumin (*n* = 14)			Placebo (*n* = 9)			*p*-Value Post-Intervention Between Groups	*p*-Value Interaction
		Pre-Intervention	Post-Intervention	*p*-Value Within Group	Pre-Intervention	Post-Intervention	*p*-Value Within Group		
Weight (Kg)		88.56 ± 21.94	88.87 ± 22.00	0.953	80.59 ± 14.69	80.74 ± 14.80	0.999	0.716	0.820
BMI (Kg/m^2^)		32.36 ± 7.33	32.48 ± 7.35	0.926	29.89 ± 4.72	29.59 ± 4.47	>0.999	0.631	0.864
Waist circumference (cm)		103.14 ± 15.23	104.50 ± 15.64	0.507	96.94 ± 8.38	96.38 ± 9.23	>0.999	0.354	0.311
Blood pressure (mmHg)	Systolic	133.88 ± 13.74	137.30 ± 14.16	0.737	115.96±11.55	117.85 ±16.83	>0.999	0.007	0.800
	Diastolic	81 (75–84.75)	84.25 (75.42–91.17)	0.938	81 (73.67–81.67)	77 (73.33–81.67)	0.667	0.467	0.231
BMC (Kg)		2.28 ± 0.45	2.27 ± 0.42	0.891	2.14 ± 0.46	2.11 ± 0.44	0.249	0.458	0.454
BMD (gm/cm^2^)		1.10 ± 0.12	1.09 ± 0.11	>0.999	1.06 ± 0.14	1.05 ± 0.12	0.620	0.881	0.500
Fat mass (Kg)		34.71 ± 12.96	35.14 ± 13.55	>0.999	30.95 ± 6.78	31.07 ± 7.29	0.646	0.975	0.614
Lean mass (Kg)		48.0(44.11–54.97)	47.73 (45.47–55.17)	0.921	50.09 (41.02–54.18)	50.84 (41.07–56.67)	>0.999	>0.999	0.732
% fat		38.38 ± 8.44	38.48 ± 8.78	>0.999	37.98 ± 5.13	38.00 ± 5.36	0.587	>0.999	0.259

All the values were reported as mean ± standard deviation. BMI: body mass index; BMC: bone mineral content; BMD: bone mineral density

## Data Availability

The 16S sequencing data have been submitted to the National Center for Biotechnology Information (NCBI) Sequence Read Archive (SRA) and are accessible under BioProject accession number PRJNA1217483. Other datasets are available from the corresponding author upon reasonable request due to privacy, legal or ethical reasons.

## Supplementary Materials

The following supporting information can be downloaded at: https://www.mdpi.com/article/10.3390/nu17132164/s1, File S1: CONSORT 2010 checklist of information to include when reporting a randomized trial.

## Abbreviations

The following abbreviations are used in this manuscript:

ALT	Alanine Aminotransferase
ANOVA	Analysis of Variance
AST	Aspartate Aminotransferase
ASVs	Amplicon Sequence Variants
BMC	Bone Mineral Content
BMD	Bone Mineral Density
BMI	Body Mass Index
CDC	Centers for Disease Control and Prevention
DEXA	Dual-Energy X-ray Absorptiometry
GCP	Good Clinical Practice
HbA1c	Hemoglobin A1c
HDL	High-Density Lipoprotein
IRB	Institutional Review Board
LDA	Linear Discriminant Analysis
LDL	Low-Density Lipoprotein
LEfSe	Linear Discriminant Analysis Effect Size
Post.CUR	Post-Curcumin
Post.Pla	Post-Placebo
Pre.CUR	Pre-Curcumin
Pre.Pla	Pre-Placebo
rRNA	Ribosomal Ribonucleic Acid
VLDL	Very Low-Density Lipoprotein
